# On the Ulcerative Process in Joints

**Published:** 1834-07-01

**Authors:** 


*On the Ulcerative Process in Joints.* By C. ASTON KEY, Surgeon of Guy's Hospital. [*Medico-Chirurgical Transactions*, Vol. XVIII. Part I.]

*A Practical Treatise on Diseases of the Joints.* By W. J. WICKHAM, Surgeon to the County Hospital, Winchester.—Winchester, 1833. 8vo. pp. 178; two Plates.

ALTHOUGH we acknowledge the wisdom of regarding science as of no country, and the philosopher as a citizen only of the world, yet we confess that we are not so thoroughly imbued with this spirit of literary cosmopolitanism as to follow with equal interest native and foreign investigations. On the contrary, we dwell with peculiar pleasure on those subjects to the elucidation of which British talent and industry have principally contributed. Amongst such as appertain to our profession, the diseases of the joints stand, "if not first, in the very first line," as originally classified by a British surgeon, and as owing every subsequent improvement, both in their pathology and treatment, (except in one instance, the use of moxa,) to British observation and experience. But it not unfrequently happens, when any pursuit has been attended by extraordinary success, that a crowd of adventurers are tempted into the same field, under the impression that their labours will be similarly rewarded; forgetting, however, that a rich harvest temporarily exhausts the soil, and that the succeeding crops (if any be obtained,) must be proportionably scanty. The same pains which, on subjects so disadvantageously situated, have yielded no returns, if expended on others hitherto untried, might, and most probably would, have been rewarded by highly valuable practical discoveries.

These observations appear to us peculiarly applicable to the works under review. The ingenuity and talent with which Mr. Key has always prosecuted his inquiries have never been so unsuccessful as in the present instance; and the persevering assiduity and zeal, of which Mr. Wickham's book affords internal evidence, could on very few subjects have been so sparingly recompensed. The errors into which these gentlemen have fallen are of a very opposite character. Mr. Wickham has detailed the result of fifteen years' experience; and, if a book on the treatment of diseases of the joints were required, the fidelity and industry with which his treatise has been executed would entitle it to the attention of the profession: but, as every surgeon is, or ought to be, provided with Mr. Brodie's work, which contains all that is valuable in Mr. Wickham's, its publication cannot be regarded as contributing to the advancement of science, though it may fulfil the author's other intention, by affording the governors of the Winchester hospital a proof of his zeal. On the other hand, Mr. Key, in his search after novelty, has indulged in speculations (to say the best of them) of very questionable value, and has supported them by a train of desultory reasoning, which it is difficult to comprehend, and much more so to analyse or abridge. We shall, however, make the attempt; and, if unable to follow him through the whole labyrinth of his theories, we may select a few passages that may serve as a specimen of his style and arguments.

It is the principal object of Mr. Key's paper to demonstrate that ulceration of cartilage is not effected by the agency of its own vessels, but that it is accomplished by the blood-vessels of the surrounding parts; that the process is modified by the cause which occasions it; that, when it arises from inflammation of the synovial membrane, a highly vascular false membrane is formed for the purpose of removing the cartilage; when from wounds of the joints, the absorption is accomplished by means of the vessels of the opposite synovial surface; and, lastly, when commencing in disease of the bone, certain vascular granulations spring up from the cancellous structure, which act on the cartilage in contact with them.

Now, it is no new theory that cartilage is destroyed by the vessels of the parts with which it is connected, but various arguments have been adduced in opposition to it: 1st, the known vascularity of cartilage during infancy; 2d, vessels are occasionally found ramifying through it during the progress of disease; 3dly, it is evidently endowed with a power of repairing the effects of the friction to which it is constantly exposed; and, lastly, it sometimes undergoes a morbid change of structure, which can only be effected by the absorption of the old and the deposition of new materials. These objections have been hitherto considered sufficient to overthrow the theory of the extrinsic absorption of cartilage; and it was therefore incumbent on Mr. Key, when attempting its revival, to commence by replying to them; but, so far is this from the course which he has pursued, that he has not even noticed their existence. We cannot, indeed, account for the author's silence on these points, since they are detailed at length by Mr. Brodie. There is, however, one novelty in Mr. Key's hypothesis,—that ulceration, whether in joints or elsewhere, is not effected by means of the absorbents, but by the extreme branches of the venous system, and he states the following as the grounds of his opinion: 1st, parts are disposed to ulcerate in proportion as they are well supplied with blood; 2dly, where absorption is going on there is always a corresponding increase of vascularity; and, 3dly, those structures in which but few vessels exist are intermingled, during the ulcerative process, with a vascular structure. The first is illustrated by the frequency of ulceration in inflamed mucous membranes; the second by the changes which take place during the ulceration of serous membranes; and the third is exemplified in the absorption of ligamentous fibre, which he thus describes:

"The ligament, instead of preserving its usual form and size, becomes distended and feels pulpy. When cut into, the fibres are found to be separated from each other by a vascular structure, which upon being injected has a villous appearance. This interstitial vascular mass is the reticular membrane that, in the healthy structure, unites the ligamentous fibres; under inflammation it becomes highly vascular, and assumes the appearance alluded to, while the fibres of the ligament retain their natural glistening appearance until, in the progress of the disease, they at length become softened and pulpy previously to their undergoing absorption. It is not improbable that the ligamentous fibres themselves are passive in the ulcerative process, which there is some reason for believing is performed entirely by the vascular tissue that surrounds them." (P. 214.)

Caution in matters of science can scarcely be too highly commended; but Mr. Key's readers would probably have pardoned him, if, after several pages of reasoning, his conclusions had been put in a rather more tangible form than that they are "not improbable," and that "there is some reason for believing" them. Nevertheless, it appears to us that Mr. Key's confidence is well proportioned to the strength of his arguments, and that he has shown the correctness of his own judgment in thus lightly estimating their force. The value of the first proposition, that there is a remarkable disposition to ulceration in those textures that are well supplied with blood, may be estimated from a very cursory examination. The choroid coat of the eye, the thyroid gland, and the spleen, are among the most vascular structures of the body, yet are they also among those least liable to ulcerate. On the other hand, if we examine what parts most readily take on this ulcerative process, we must enumerate the cornea (the least vascular tunic of the eye), the cartilages (the parts of the articulations which are most scantily supplied with blood), and the cicatrices of former sores which possess but an imperfect organization.

Mr. Key has not been more fortunate in his selection of the mucous membranes as an illustration of this position. The conjunctiva palpebralis, which is a highly vascular mucous membrane, does not by any means display this disposition to ulceration; and every surgeon must know that the mucous membrane of the urethra frequently suffers considerable violence and laceration from the unskilful use of bougies, while ulceration of this surface is comparatively of rare occurrence. It is undoubtedly true that the lining membrane of the alimentary canal is very liable to ulcerate; but this statement, considered as an argument, is open to an objection which Mr. Key has foreseen, and to which he has endeavoured to reply in the following passage:

"The action of the veins in producing ulceration of a villous surface, as that of the intestinal canal, is by no means rendered improbable by the membrane being abundantly furnished with absorbents. Gendrin mentions that in those who have died with ulceration of the intestine, he has usually found the veins either filled with pus, or inflamed upon their inner surface. The same author relates an experiment of injecting pus into the pleural cavity of an animal, and at the expiration of twenty-four hours finding, on dissection, a considerable quantity of the fluid in the branches of the thoracic veins. Other observations might be adduced in support of the opinion that the function of the absorbents is confined to nutrition, to the removal of interstitial fluids, and to the preservation of the form of the body during growth, or, as Mr. Hunter has termed it, modelling absorption; and that progressive absorption or ulceration is effected through the agency of the extreme branches of the venous system." (P. 211.)

No expressions could possibly indicate a greater degree of doubt than those with which he commences; yet, within ten lines, this doubt has risen to an opinion, not simply, that veins may absorb, but that the whole ulcerative process is effected through their agency; and, when we examine the arguments that have had so much power on Mr. Key's mind, our surprise at their effect is rather augmented than diminished: for, though pus has been found in the veins, it is, according to our author's own statement, not always there; hence the action of the veins and the ulcerative process cannot be connected as cause and effect. Besides, pus has not unfrequently been found in the absorbents; a fact which strongly militates against the theory that they are only concerned in modelling absorption. The "other observations" to which Mr. Key alludes are, we presume, to be found in a note containing an experiment, which we subjoin.

"Professor Coleman, of the Veterinary College, at my request, made the following experiment. He caused to be inserted in the inner part of the thighs of an ass a rowel, which, at the expiration of four days, had established a copious suppuration. On the fourth day a small quantity of prussiate of potass was inserted in each sore, and allowed to remain six hours, at the end of which period the animal was killed. To ascertain which of the two systems, the venous and the absorbent, had taken up most of the *salt*, I removed some blood from the iliac veins on both sides, and some from the mesenteric veins; and Mr. Coleman's dissector collected half an ounce of fluid from the thoracic duct. These I submitted to Mr. Alfred Taylor, our lecturer on medical jurisprudence and chemistry, who favoured me with the following analysis. 'No. 1, the blood from the iliac veins, contains the prussiate in large proportion. No. 2, the serum from the thoracic duct contains it in about the ratio of $\frac{1}{50}$ of No. 1; and No. 3, the blood from the mesenteric veins, after standing six days, shews evidence of the prussiate in the ratio of about $\frac{1}{50}$ of No. 2, and therefore, of about $\frac{1}{2500}$ of No. 1." (P. 212, *note.*)

It is generally believed that the absorbents communicate with the veins during their whole course; but we need not remind so excellent an anatomist as Mr. Key, that there is a distinct absorbent trunk opening into the iliac vein. The presence, therefore, of the prussiate of potass in these veins demonstrates nothing, since it might have found admission by this absorbent vessel; and the minuteness of the portion found in the mesenteric veins only corroborates what might have been anticipated, viz. that a part of the salt had been eliminated during the progress of the blood through the body. Thus, all that this experiment can be said to prove decisively is, that the absorption is partially, if not wholly, carried on by the absorbents,—the very conclusion which it is intended to controvert. By a very slight modification of the same line of argument, we might expose the futility of Mr. Key's next position, viz. that "all structures, previously to being removed by ulceration, become unusually vascular, as if a more complete development of the sanguineous tissue were essential to this mode of absorption," and reduce it to its old-fashioned form, that ulceration is one of the sequelæ of inflammation.

Ere we quit this part of our subject, let us be careful not to be misunderstood. We do not mean to deny the action of the veins in absorption; we mean only to dispute the sweeping proposition of Mr. Key, that ulceration is effected through their agency alone, and to show that the arguments which he employs are not entitled to the importance which he attaches to them, while he totally neglects to notice others which lie on the surface, but which militate against his opinion. What can be more striking than the phenomena attendant on the absorption of certain poisons?—syphilis, for instance. It has never fallen to our lot to witness inflammation of the veins of the penis from the irritation occasioned by the passage of the virus: but a chancre and a bubo have been almost as closely associated in our mind as a sound with its echo; and we should as soon have dreamed of doubting the connexion between the former as between the latter.

We now follow Mr. Key to his exposition of the doctrine of passive absorption of cartilage; and his reasoning upon this subject is, if possible, more inconclusive than on the former.

Having stated the appearances presented during the absorption of ligamentous fibre, which we have before quoted, and which we may now paraphrase into a form under which our readers may perhaps recognise an old acquaintance, viz. that when any part, consisting of two different structures, is inflamed, that structure which is most vascular in health will be the most injected during disease:—having stated this, Mr. Key proceeds to describe the absorption of dead bone in necrosis, and to draw an analogy between this process and that of the absorption of cartilage. Now, had Mr. Key been satisfied with informing us, that "the dead bone, having no power of self-removal, the surrounding parts are called upon to perform the office of removing the useless mass," none of his readers could have accused him of a taste for innovation, and we for our parts should have so far assented to its truth as to rank it amongst self-evident truisms; but, when our author proceeds to argue that, because dead bone cannot perform any vital action, therefore it is probable that such vital actions cannot be performed by living cartilage, far from being able to trace an analogy, we rather recognise an antithesis, of circumstances.

We turn, however, with no small degree of pleasure from Mr. Key theoretical to Mr. Key practical; and we wish that we were able to devote a larger portion of our review to this part of the subject. His descriptions of the appearances presented in post-mortem examinations of diseased joints are extremely vivid, and evince the hand of a master. The following passage will excuse us for quarrelling with Mr. Key's theories, since they have occupied much of his paper, and cheated us, perhaps, of many such descriptions.

"The first circumstance that strikes us, on opening a diseased joint, is, the different degrees of ulceration in the articular surfaces, and the different extent to which the interarticular cartilage and ligaments have suffered. This will depend upon the part in which the diseased action has commenced, which perhaps in most cases is determined by accident, as the seat of the blow or sprain which may have excited the inflammation, or the form of the joint producing unequal bearing upon the surfaces, and thus determining the inflammation to that part where the pressure is greatest. The inner part of the knee joint usually exhibits the most extensive ulceration on account of the oblique bearing of the femur, and its consequently unequal pressure on the inner part of the head of the tibia. We therefore find the inner semilunar cartilage more often destroyed than the outer, and a corresponding destruction of the cartilage covering the inner condyle of the femur and inner part of the head of the tibia. The patella and the extremity of the femur are the parts on which the ulcerative process can be best traced on account of the disease being in these less advanced. In the former bone the first part that commonly gives way to ulceration is the margin of the cartilage, where the synovial membrane is reflected from it. At this point sulci of different depths are formed which cannot be always distinguished, until the thickened edge of the synovial membrane is raised. The ulcerated surface sometimes exhibits parallel vascular lines verging towards the centre, and having their origin from the synovial membrane. The synovial membrane at this part, if the vessels are well filled with fine injection, appears highly vascular, and fringed or villous like a mucous membrane. This increased vascularity is particularly noticeable at the edge of the membrane, and in these portions of the fringed margin that correspond to the ulcerated surface of the cartilage; the other parts of the synovial membrane have their vascularity but slightly increased. This highly vascular fringe of membrane is a newly organized, and will be found in some parts to be a superadded, structure, for the purpose of producing ulceration of the contiguous cartilage. It may when recently formed be raised in some parts from the synovial membrane, but is found to adhere very slightly to that part of the cartilage where ulceration is going on; this adhesion is not perceived unless the joint is opened with care." (P. 222.)

In describing the progress of ulceration of the cartilage of the hip-joints, Mr. Key states his belief that it commences more frequently in the ligamentum teres than is generally supposed; but, as he gives due credit to Mr. Brodie's assertion, that it does exist as a primary disease, so far from controverting his opinion, we shall quote it, for the instruction of our readers.

"The beginning of the affection is frequently to be traced to a fall, by which the legs have been forcibly separated, and the ligamentum teres stretched. In some cases, the injury has been so considerable, as to occasion the patient to rest the limb for some days, on account of the severity of the pain. This to a certain extent subsides, and the inflammation that remains assumes the chronic form. If the patient's health is good, he recovers with only a slight temporary weakness in the joint; in the more feeble habit, with a tendency to strumous action, the disease gradually passes into the ulcerative form. Sometimes, from the tender age of the child, no cause can be assigned for the disease; perhaps, in some instances, it may have a purely constitutional origin. The motions of the joint, that give the patient most pain, are strongly indicative of the seat of the affection; in the earliest stage, before the soft parts could well be affected, if the disease commenced in the cartilage, eversion of the thigh, and abduction of the limb from the other, produce the greatest degree of suffering to the patient; while he can bear the joint to be flexed, and to be slightly inverted, without complaining. A similar indication of the ligamentum teres being inflamed, is the pain sometimes expressed on pressing the head of the femur against the acetabulum; in its healthy state the ligament, being lodged in the hollow of the acetabulum, receives but little pressure; but when it is swelled by inflammation, the cavity of the joint affords it less protection, and when pressure is made by forcing the head of the femur upwards, the ligament is compressed, and usually produces some degree of pain. The circumstance, too, of the ligamentum teres being destroyed by ulceration, when the head of the bone and acetabulum are only partially ulcerated, may be considered as presumptive proof of it being very early engaged in the disease. There are few cases of post mortem inspection of the hip-joint in an advanced stage of disease, in which the ligamentum teres is not found destroyed." (P. 231.)

We must here terminate our quotations. We could have wished that Mr. Key had more carefully revised his paper before he consented to its publication; for his arrangement might have undergone considerable improvement, and some important errors might have been corrected. He states, for example, that there are four methods in which ulceration takes place in cartilage, and details but three, substituting for the fourth an account of its conversion into a fibrous structure. He would probably, too, have thought it right to suppress certain parts, in which he expresses a most unsatisfactory degree of doubt. Thus, in a single page, we find the following hesitating expressions: "There are some circumstances that tend to throw some doubt;" "it is by no means satisfactorily ascertained;" "is still less definitively understood;" "it yet remains a problem;" and "is by no means rendered improbable:" besides one or two others, which betray but little more confidence.

In common with all our professional brethren, we feel under obligations to Mr. Key, for his ardent pursuit of science, and his readiness to communicate whatever appears to him to be important; but, we regret that a surgeon, possessing such ample opportunities of watching disease, such accuracy of observation, and such powers of description, should display so great a predilection for hypothesis. We would venture to remind Mr. Key, that the cause of science is more advanced by the publication of a single fact than by a whole volume of theory; that men of the most transcendent talent have not deemed it beneath them simply to detail what their eyes have seen and their hands have handled: and we desire to recall him to that course of strict investigation which has been consecrated by their invaluable discoveries.

We have devoted so much space to Mr. Key, that we can afford but little to Mr. Wickham; but this is of the less consequence, as he lays no claim to novelty for his opinions, and there is, consequently, no room for discussion. His arrangement of his subject is simple, and it is so far good, though he has left so many points untouched, that his work would be better named a Treatise on *some* of the Diseases of the Joints. Meanwhile he has introduced certain topics rather unnecessarily: for instance, he commences with an account of various kinds of exostoses in the vicinity of the articulations. Now, as these have no connexion with the structures of which the author professes to treat, excepting in as far as they impede their motion, he might with equal propriety have introduced an account of popliteal aneurism, or of glandular abscess in the axilla. Caries of the bones, and necrosis, are in a great measure foreign to his subject; and it appears to us that such little notice as it might be requisite to take of them would more properly have been stated in connexion with their effects on the joints. Again, the author considers the scrofulous affections of the bones, ultimately causing absorption of the cartilage, as synonymous with necrosis and caries: be this as it may, (and we are inclined to differ with Mr. Wickham,) yet the difference of treatment required ought to have secured for them a separate consideration.

In reviewing a work, it is a good practice to select a passage, in order that the reader may judge for himself of the style in which it is written; and, as we have devoted the former part of this article to the pathology of ulceration of the cartilage, we shall quote Mr. Wickham's account of the symptoms of that disease during life. It forms a good specimen of the merits of the whole work, as the author has evidently bestowed more of his attention on the means of detecting disease, and the method of curing it, than on any curious questions concerning its nature and causes.

"Primary ulceration of the cartilage is characterized by strongly marked symptoms, which differ essentially from those which accompany diseased action in other parts of the joint. They are such as satisfactorily to lead to a detection of the actual seat of the disease.

"The pain is peculiar: it is continued, and of the pricking kind. Patients describe it by saying they feel as if an animal was gnawing at the part. This pain is deep in the articulation; but there is for the most part a remote or distant pain, a dull and continued aching down the lower part of the limb. This pain, although generally extending along the whole, now and then confines itself to one particular spot. Even then the pain is the same, resembling that of rheumatism. When the cartilage of the hip joint is inflamed, the distant pain is particularly along the outer side of the thigh, knee, and legs; but it is most severe in the knee and ankle. In disease of the cartilage of the shoulder, the pain extends along the outer side of the arm to the elbow, and onwards to the two last fingers. The distant pain is generally accompanied by a slight degree of numbness, and in very irritable persons by cramps or spasmodic twitchings of the large muscles of the limb. This is a great aggravation of suffering, and renders it scarcely tolerable. The pain, both that which is in the joint and that which is at a distance, is aggravated by pressure or forcing one bone against another; and it is curious to observe, when the pain of the joint has been in less degree than the remote pain, how decidedly pressure produces the distant pain: for example, I have repeatedly found pressure on the groin revive the great suffering which the patient has been affected with in the knee, in cases of disease of the cartilage of the hip.

"*Absence of Swelling.*—In the first stages of this disease no swelling of the joint is observable. It is only when the inflammation has extended to the other textures that swelling occurs. The distinctions, then, by which disease of this structure is to be known, are the peculiarity of pain, and the absence of swelling. These are the only symptoms of the first stages of the attack, and do not vary until the inflammation has proceeded to other parts; when, however, this does take place, new symptoms arise, and are such as are attendant on disease commencing in the other textures. There is one symptom which may be perceived in the latter stages of ulceration of the cartilage, which cannot be discovered until absorption has gone to a considerable extent. I mean a grating sensation by friction of one bone against another. To detect this, it is necessary that a surgeon should be familiar with the peculiar sensation communicated, and should be able to distinguish it from that of fracture within the joint, or the crackling of certain states of bursal inflammation. Primary ulceration of the cartilage happens more frequently in subjects which partake of a scrofulous habit of body than in other constitutions. I do not mean to say that the disease is in the least degree to be identified with scrofula; but it appears most commonly in feeble and delicate constitutions, and particularly in those persons who have been subject to glandular affections of a scrofulous character. It appears commonly in children, or those about the age of puberty. It is comparatively of rare occurrence in persons in after-life. For the most part no cause can be assigned for its coming on; still it sometimes may be traced to injuries or concussions which the joint may have received, the constitution or part not having power or activity to repair the mischief. This affection is very frequently preceded by fevers, following, after the manner of abscesses, close on the convalescence from any severe febrile disorder. Debility of constitution, whether natural or induced by previous complaint, is favourable to the occurrence of this complaint. That ulceration of the cartilage appears as a constitutional affection is very manifest, from the circumstance that a disposition exists in some persons to take on this disease in several joints at the same time, without sufficient local exciting cause to bring it into action." (P. 49.)

In his treatment of this affection, Mr. Wickham agrees with every good practical surgeon, as to the importance of absolute rest. His observations on this subject are worthy of quotation.

" In all cases of diseased action going on within the articulation, but more particularly in inflammation of the cartilage, rest is to be considered the most important and efficient means of arresting its progress. It is important, because no other remedy can be serviceable without perfect quietude; and it is efficient, inasmuch as it is of itself oftentimes enough to effect a cure. It is a well known fact that an inflamed surface cannot recover itself so long as it is suffering under the continuance of an irritating cause. A wound will not be cured so long as its surface is rubbed, and the efforts at cicatrization are thereby prevented. Let us apply this reasoning to the condition of the articulating cartilage under ulceration. Every motion of one bone upon another must by friction irritate the inflamed and ulcerated cartilage, and, moreover, where a healing process has actually commenced, it is entirely undone by a single movement. It is therefore to be kept in view, that it is not only necessary to preserve the joint at rest in order to remove the irritating cause and subdue the inflammation which has begun, but also to prevent the whole respiratory process which has been in operation from being rendered fruitless. On the first appearance of symptoms, then, which indicate inflammation of the cartilage, I confine the joint to a perfect quietude by means of a pasteboard splint, and such trappings as may effectually secure the joint from motion, or from the least friction on the diseased surface. I bear in mind also, that the first act of inflammation in the cartilage is its destruction, and that it is necessary to continue the restraint until the ulcerated surface may have become healed. For want of this precaution, and the surgeon delaying his permission of motion, I have witnessed much mischief in the relapse of the case; and this especially when the patient is to be removed from the vigilant eye of the surgeon." (P. 53.)

We are not friendly to endless subdivisions of diseases; they rather confound than elucidate the subject: but Mr. Wickham appears to have fallen into the opposite error, and has not admitted even those divisions of his subject which are valuable in practice. In addition to the instance of scrofulous disease before mentioned, we observe that, under the head of acute idiopathic inflammation of the synovial membrane, he classes the rheumatic and gouty affections of the joints, without noticing the difference of treatment which they require.

If we were inclined to quarrel with Mr. Wickham, we should complain of certain rather lengthy arguments to refute errors long since abandoned. The term *spina ventosa*, with the absurd theory on which that name was founded, has long since peacefully slept with its fathers; but our author has again called it into being, apparently for no other purpose than to repeat the pleasure of destroying it.

Again, when treating of the supposed elongation of the limb in incipient disease of the hip-joint, he offers the following explanation of its cause, and then proceeds to demonstrate its correctness.

"The deception is, I conceive, produced by the position into which the limb is thrown by rigid muscular action. It will be observed that the leg is projected away from its fellow, and the toe is turned a little outwards, so that a person comparing the two legs will bring the sound towards the affected, rather than by great force and consequent pain draw the inflamed limb towards the sound x 2

one. Thus the sound leg does not traverse the median line without losing from an inch and a half to two inches of its length." (P. 157.)

Now, it has not fallen to our lot to see such a clumsy expedient put into practice; a piece of tape does away with the error, and the necessity of an explanation.

But the taste of searching out errors is as useless as it is unpleasant. The general impression which the perusal of a book leaves upon the mind is what is really valuable: our impression, then, in closing Mr. Wickham's book, is, that its author is a very sensible practitioner, who has thought much on professional subjects, and who has faithfully recorded his experience, which is valuable as additional testimony in corroboration of Mr. Brodie's statements; and though we cannot flatter him that his treatise will be generally perused while Mr. Brodie's work exists, yet it will certainly add to his reputation as a sound and scientific surgeon.

